# Evolutionary Analysis of the *LAFL* Genes Involved in the Land Plant Seed Maturation Program

**DOI:** 10.3389/fpls.2017.00439

**Published:** 2017-04-04

**Authors:** Jing-Dan Han, Xia Li, Chen-Kun Jiang, Gane K.-S. Wong, Carl J. Rothfels, Guang-Yuan Rao

**Affiliations:** ^1^School of Life Sciences, Peking UniversityBeijing, China; ^2^RDFZ XiShan SchoolBeijing, China; ^3^Department of Biological Sciences, University of Alberta, EdmontonAB, Canada; ^4^Department of Medicine, University of Alberta, EdmontonAB, Canada; ^5^BGI-Shenzhen, Beishan Industrial ZoneShenzhen, China; ^6^University Herbarium and Department of Integrative Biology, University of California, BerkeleyCA, USA

**Keywords:** seed maturation program, LAFL network, gene structure, expression analysis, phylogenetic analysis

## Abstract

Seeds are one of the most significant innovations in the land plant lineage, critical to the diversification and adaptation of plants to terrestrial environments. From perspective of seed evo-devo, the most crucial developmental stage in this innovation is seed maturation, which includes accumulation of storage reserves, acquisition of desiccation tolerance, and induction of dormancy. Based on previous studies of seed development in the model plant *Arabidopsis thaliana*, seed maturation is mainly controlled by the LAFL regulatory network, which includes *LEAFY COTYLEDON1* (*LEC1*) and *LEC1-LIKE* (*L1L*) of the *NF-YB* gene family, and *ABSCISIC ACID INSENSITIVE3* (*ABI3*), *FUSCA3* (*FUS3*), and *LEC2* (*LEAFY COTYLEDON2*) of the B3-*AFL* gene family. In the present study, molecular evolution of these *LAFL* genes was analyzed, using representative species from across the major plant lineages. Additionally, to elucidate the molecular mechanisms of the seed maturation program, co-expression pattern analyses of *LAFL* genes were conducted across vascular plants. The results show that the origin of *AFL* gene family dates back to a common ancestor of bryophytes and vascular plants, while *LEC1*-type genes are only found in vascular plants. *LAFL* genes of vascular plants likely specify their co-expression in two different developmental phrases, spore and seed maturation, respectively, and expression patterns vary slightly across the major vascular plants lineages. All the information presented in this study will provide insights into the origin and diversification of seed plants.

## Introduction

Seeds, as propagules and dispersal units, play very important roles in the adaptation of seed plants to terrestrial environments (Kenrick and Crane, 1997; Becker and Marin, 2009; Radoeva and Weijers, 2014). Seed development is an intricate process, which can be divided into two conceptually distinct phases: embryo morphogenesis and seed maturation (Goldberg et al., 1994; Harada, 1997; Gutierrez et al., 2007). Seed maturation, which includes all of the events occurring after cell division has ceased within the embryo (following Harada, 1997), can be considered as a developmental module that is added after embryogenesis. It is accomplished with the accumulation of nutrient reserves, the acquisition of desiccation tolerance, the desiccation of seeds, the suppression of precocious germination, and the induction of dormancy (Goldberg et al., 1994; Harada, 1997); these features are each thought to be important in the adaptation of plants to variable and harsh terrestrial environments. Overall, it was considered that seed maturation is a more recently derived adaptation program of land plants (Harada, 1997; Santos-Mendoza et al., 2008).

According to previous studies, especially of Arabidopsis, the seed maturation program involves complex regulatory networks that regulates a large set of genes (Verdier et al., 2013; Righetti et al., 2015). The LAFL network is one of those regulatory networks, which includes *LEAFY COTYLEDON1* (*LEC1*) and *LEC1-LIKE* (*L1L*) of the *NF-YB* gene family, and *ABSCISIC ACID INSENSITIVE3* (*ABI3*), *FUSCA3* (*FUS3*), and *LEC2* (*LEAFY COTYLEDON2*) of the B3-*AFL* gene family (Suzuki et al., 1997; Luerßen et al., 1998; Santos-Mendoza et al., 2008; Suzuki and McCarty, 2008; Swaminathan et al., 2008; Xie et al., 2008; Jia et al., 2013; Kirkbride et al., 2013). On a basic level, this network was thought to orchestrate the accumulation of storage compounds and the acquisition of desiccation tolerance in seed maturation (Harada, 1997; Santos-Mendoza et al., 2008; Jia et al., 2013; Radoeva and Weijers, 2014). Meanwhile, the LAFL network also represses the expression of genes required for the transition from embryonic to vegetative developments, i.e., the suppression of precocious germination (Giraudat et al., 1992; Nambara et al., 1992; Stone et al., 2001).

Four conserved protein domains can be recognized in the B3-*AFL* gene family regulatory factors of *Arabidopsis thaliana*, designated A, B1, B2, and B3 (Giraudat et al., 1992; Suzuki et al., 1997). The A-domain is a functional acidic activation domain found at the N-terminal (McCarty et al., 1991). The B1-domain consists of about 30 amino acids (AAs) involved in the physical interaction with the bZIP transcription factor, such as *ABI5* (*ABSCISIC ACID INSENSITIVE5*; Nakamura et al., 2001). The B2-domain consists of about 15 AAs, which have been shown to be responsible for the ABA-dependent activation of ABA-regulated genes, through the ABA-response element (ABRE; Hill et al., 1996; Bies-Etheve et al., 1999; Ezcurra et al., 2000). The B3-domain, composed of about 100 AAs, has been shown to act as the DNA binding domain (Suzuki et al., 1997; Nag et al., 2005). For the *AFL* family genes, *ABI3* has all the recognized domains of this gene family (Giraudat et al., 1992; Suzuki et al., 1997). *FUS3* contains the A, B2, and B3 domains, but the A-domain in the C-terminal (Lu et al., 2010). *LEC2* has only the B2 and B3 domains. In the monocots, there are different names for *AFL* genes. For example, five *AFL* gene homologous were found in *Oryza sativa*, e.g., *OsVP1, OsLFL1*, and *OsIDEFs*. *OsVP1*, which contains A, B1, B2, and B3 domains, is homologous with Arabidopsis *AtABI3* (Hattori et al., 1994), and *OsLFL1* is homologous with Arabidopsis *AtFUS3* (Peng et al., 2008). Another three *OsIDEFs* are considered to be *AtLEC2* type genes, but the relationship among them remains unclear (Kobayashi et al., 2007; Sreenivasulu and Wobus, 2013).

In Arabidopsis, *AFL* genes are mainly expressed in embryo development, but at different developmental stages. *AtLEC2* is expressed at early stages of embryogenesis, while *AtABI3* and *AtFUS3* are highly expressed at late stages (Stone et al., 2001; Kroj et al., 2003; Gazzarrini et al., 2004; Tsuchiya et al., 2004; To et al., 2006; Santos-Mendoza et al., 2008; Fatihi et al., 2016). According to studies in other plants, the *AFL* family genes are generally expressed in reproductive organs. For instance, *OsLFL1* is expressed exclusively in spikes and young embryos (Peng et al., 2008). In *Zea mays, ZmaAFL* genes are preferentially expressed in pollen and caryopses (Grimault et al., 2015), and in *Chamaecyparis nootkatensis*, a gymnosperm species, its *CnABI3* was detected in the megagametophytes and mature dormant embryos (Zeng and Kermode, 2004).

*LEC1*-type (*LEC1* and *L1L*) genes are of the intron-less type of the *NF-YB* family, which are derived from the intron-rich ones, and their earliest occurrence appears to be in a common ancestor of vascular plants (Yang et al., 2005; Xie et al., 2008). *LEC1* and *L1L* genes are highly expressed in embryonic cells and extra-embryonic tissues during seed development (Lotan et al., 1998; Kwong et al., 2003). Expression and function analyses of *LEC1* homologs in other species indicate that *LEC1* is essential for seed maturation (Stephenson et al., 2007; Cao et al., 2011; Salvini et al., 2012; Tang et al., 2015). In seedless vascular plants (lycophytes and ferns), the expression of *LEC1* is restricted to reproductive structures. In *Selaginella moellendorffii* (a lycophyte), high expression of *SmoLEC1* was found in strobili, where megasporangia and microsporangia are located (Kirkbride et al., 2013). Additionally, the maximal expression of *AcaLEC1* was detected in mature sporangia of the fern *Adiantum capillus-veneris* (Fang et al., unpublished data).

Complex interactions between the *LAFL* genes were found in *Arabidopsis*. For instance, the expression of *LEC1* can activate *ABI3, FUS3*, and *LEC2*, whereas the ectopic expression of *LEC2* up-regulates *LEC1* activity in vegetative tissues (Kagaya et al., 2005b; Stone et al., 2007; Guo et al., 2013). The function of *LAFL* genes involves many aspects of seed maturation including seed storage protein (SSP), late-embryogenesis- abundant (LEA) proteins, hormone metabolism, and signaling pathways (Parcy et al., 1994; Nakamura et al., 2001; Kagaya et al., 2005a,b; Alonso et al., 2009; Yamamoto et al., 2009).

The LAFL network is crucial for seed maturation, and great efforts have been made to investigate the functions of this network genes in *Arabidopsis*, but little attention was paid to the evolution of the network as a whole. With the increased availability of genomic data and a refined understanding of the distribution of *LAFL* genes, this work is now feasible. To better understand the origin and evolution of *LAFL* genes, we performed phylogenetic analyses on an extensive dataset of *NF-YB* and *AFL* gene family sequences, focusing particularly on previously underrepresented groups, such as algae, bryophytes, monilophytes, and “early diverging” angiosperms. In addition, we analyzed expression patterns of the LAFL network using online databases and our newly generated qRT-PCR data from *S. moellendorffii* and *A. capillus-veneris* (representing lycophytes and monilophytes, respectively). With these data, coupled with aforementioned phylogenetic analyses and *cis-*element information, we elucidate the evolution of *LAFL* genes and their association with the seed maturation program.

## Materials and Methods

### Gene Family Datasets

*LAFL* genes belong to two gene families: the *NF-YB* gene family and the *AFL* gene family, where the latter is a member of the B3 superfamily. To build our dataset of *AFL* genes, we first queried the Pfam database^1^ for B3 superfamily genes from three chlorophytes (*Volvox carteri, Chlamydomonas reinhardtii*, and *Chlorella variabilis*), one moss (*Physcomitrella patens*), one lycophyte (*S. moellendorffii*), and six flowering plants (*Brachypodium sylvaticum, Oryza sativa, Zea mays, Populus trichocarpa, Glycine max*, and *A. thaliana*; Supplementary Table S1); this search resulted in 730 sequences. Then, for a better understanding of the evolution of the *AFL* gene family specifically, we BLASTed the coding sequences of Arabidopsis *ABI3, FUS3* and *LEC2* against four primary sources: Phytozome^2^, ConGenIE^3^, the *Klebsormidium flaccidum* Genome Project ^4^ (Hori et al., 2014), and the OneKP database ^5^. These queries yielded 253 sequences spanning 68 species representing all major lineages of land plants. The retrieved sequences generally span the complete coding region, but some lack a few AAs at either end. The retrieved sequences range from 200 to 800 AAs in length (Supplementary Table S2).

To obtain sequences of the *NF-YB* gene family, we BLASTed Arabidopsis *LEC1* and *L1L* coding sequences against five primary sources: NCBI (National Center for Biotechnology Information ^6^), Phytozome^2^, ConGenIE^3^, the *Klebsormidium flaccidum* Genome Project^4^, and the OneKP project^5^. In total, 263 sequences spanning 29 species were collected, ranging from 100 to 300 AAs in length (Supplementary Table S3).

### Sequence Alignment

All alignments were performed at AA level. For the phylogenetic analysis of the B3 superfamily, only the B3 domain was used for alignment. For the *NF-YB* and *AFL* gene families, full-length protein sequences were used. These sequences were aligned with the MAFFT webserver (^7^Katoh and Standley, 2013). Based on sequence characteristics, we selected an alignment strategy of FFT-NS-i (*NF-YB* gene family), FFT-NS-1(B3 superfamily), and E-INS-i (*AFL* gene family), respectively.

### Phylogenetic Analysis

**FIGURE 1:**
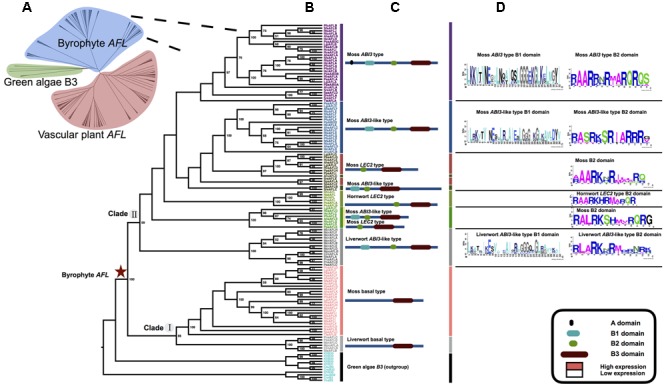
**Phylogenetic relationships of plant *AFL* gene family and the details of bryophyte. (A)** ML unrooted tree of the plant *AFL* gene family comprising 264 sequences from 72 taxa (**Table 1**; for species names see Supplementary Table S2). **(B)** ML rooted tree of bryophyte *AFL* using green algae B3 sequences as outgroups (black). Numbers on the branches indicate bootstrap values calculated from 1,000 replicates. Only values higher than 50% are shown. **(C)** Domain structure of each clade and type, two clades divided into 10 types in total. **(D)** Amino acids (AAs) characteristic of the B1 and B2 domain. The sequence logo was generated using WebLogo (weblogo.berkeley.edu/).

**FIGURE 2:**
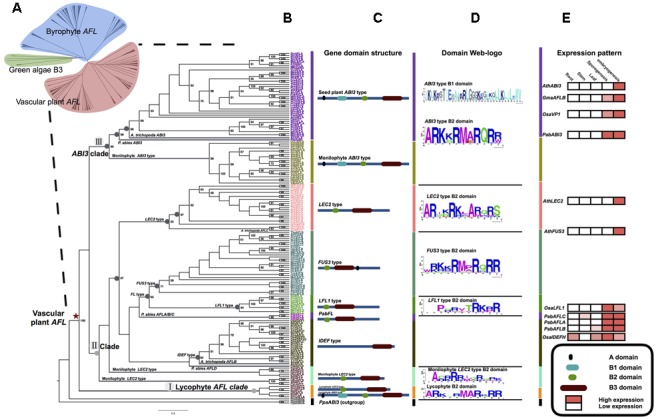
**Phylogenetic relationships of plant *AFL* gene family and the details of the part of vascular plant. (A)** ML phylogenetic relationships of plant *AFL* gene family comprising 264 sequences from 72 taxa (**Table 1** for species names see Supplementary Table S2). **(B)** ML rooted tree of vascular plant *AFL* using *PpaABI3* sequences as outgroups (black). Numbers at the branches indicate bootstrap values calculated from 1,000 replicates. Only values higher than 50% are shown. **(C)** Domain structure of each clade and type, three clades divided into 10 types in total. **(D)** AAs characteristic of the B1 and B2 domain. The sequence logo was generated using WebLogo (weblogo.berkeley.edu/). **(E)** Expression pattern analysis of several *AFL* genes; expression levels are calculated using the database GENEVESTIGATOR, ConGenIE (Supplementary Figure S3), and data from previous studies (Zeng et al., 2003; Zhang and Xue, 2013).

The final alignments were analyzed using Protest (Abascal et al., 2005) to choose the best-fitting AA model; the JTT + I + G substitution model was selected for all alignments according to the AIC and BIC selection criteria. Maximum likelihood (ML) phylogenetic analyses were performed with RaxML (Stamatakis et al., 2008) and evaluated by the bootstrap method using 1000 replicates. Trees were observed and edited for presentation using FigTree v1.4.2. ^8^ Based on phylogenetic reconstruction of the B3 superfamily (Supplementary Figure S1), we re-built a dataset with an ingroup sample of 253 *AFL* genes, and an outgroup of 11 B3 genes from four algal species for further phylogenetic analysis of *AFL* gene family (**Figures 1A, 2A, Table 1**, and Supplementary Table S2). For bryophytes and vascular plants, further phylogenetic analyses were carried out, respectively (**Figures 1B, 2B**). In addition, phylogeny reconstruction of the *NF-YB* family was performed using the data set containing 263 sequences of 29 species with whole genome sequences (Supplementary Figure S3). To explore the relationship of *LEC1*-type genes and *NF-YB* family genes in non-vascular plants, 65 sequences of 26 species were used for further phylogenetic analysis (**Table 1** and **Figure 4**).

**Table 1:** Sampling in *LAFL* genes phylogenies.

			Classification	*AFL* species (sequences)		*LEC1*-type species (sequences)	
		Green algae	Chlorophyta	3 (5)	B3	2 (2)	NF-YB
			Charophyta	1 (6)	B3	1 (1)	NF-YB
Land plant (embryophytes)	Non-seed plant	Bryophyte	Moss	9 (66)	AFL	2 (10)	NF-YB
			Liverwort	4 (9)	AFL	1 (2)	NF-YB
			Hornwort	5 (6)	AFL	1 (1)	NF-YB
		Pteridophyte	Lycophyte	3 (6)	AFL	1 (5)	NF-YB
			Monilophyte	11 (30)	AFL	1 (5)	NF-YB
	Seed plant	Gymnosperm	Conifer	1 (5)	AFL	1 (2)	LEC1-type
		Angiosperm	Basal	1 (5)	AFL	2 (2)	LEC1-type
			Monocot	7 (35)	AFL	5 (10)	LEC1-type
			Eudicot	27 (91)	AFL	9 (25)	LEC1-type
			**Total numbers**	**72 (264)**		**26 (65)**	

### Gene Structure and *Cis*-Elements Analysis

**FIGURE 3:**
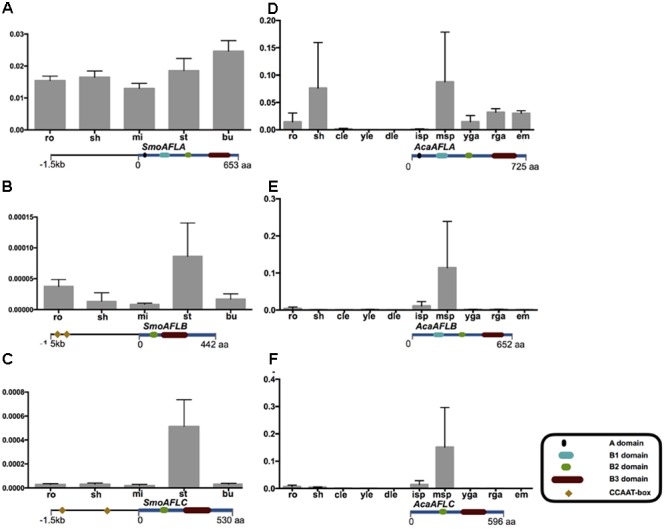
***AFL* mRNA levels in various organs of non-seed plants examined by qRT-PCR.** The CCAAT-box *cis*-elements of the promoter 1.5 kb region of three *SmoAFL* genes are highlighted in yellow blocks with an arranged number. *Selaginella moellendorffii* **(A–C)**, ro, roots; sh, shoots; mi, microphylls; st, strobili; bu, bulbils. *Adiantum capillus-veneris* **(D–F)**, ro, roots; sh, shoots; cle, curled leaves; yle, young leaves; dle, developed leaves; isp, immature sporangia; msp, mature sporangia; yga, young gametophytes; rga, reproductive gametophytes; em, embryos. The detail of each gene domain structure see Supplementary Figure S4.

**FIGURE 4:**
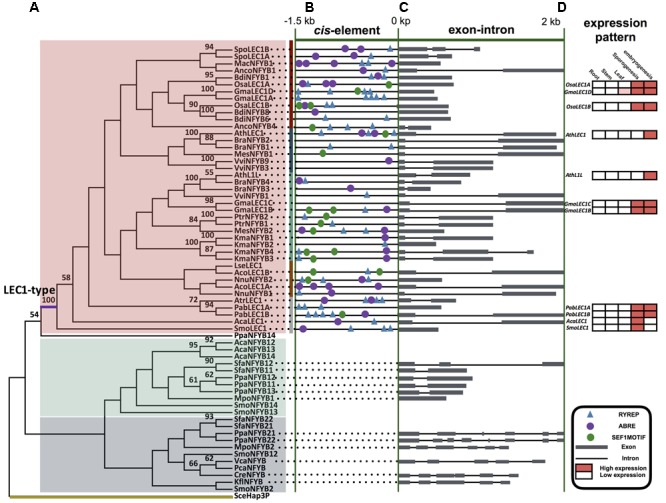
***LEC1*-type and non *LEC1*-type genes of *NF-YB* gene family. (A)** ML tree of plant *LEC1*-type and non *LEC1*-type genes comprising 66 sequences from 27 taxa (for species names see Supplementary Table S3). Numbers on the branches indicate bootstrap values calculated from 1,000 replicates. Only values higher than 50% are shown. Non-seed plant intron-rich clade, non-seed plant intron-less clade, and *LEC1*-type clade are color-coded with gray, green, and red, respectively. **(B)** The conserved *cis*-elements in the promoter 1.5 kb region of *LEC1*-type genes. Details are shown in Supplementary Table S5 with the number and sequence of motifs. **(C)** Exon-intron changes at several *LEC1*-type and non *LEC1*-type genes. Details are shown in Supplementary Table S5. **(D)** Expression pattern analysis of several *LEC1*-type genes; expression levels are calculated using the database GENEVESTIGATOR, ConGenIE (Supplementary Figure S3), and data from experimental studies (Kirkbride et al., 2013; Fang et al., unpublished data).

For the *AFL* family genes, we characterized their AA composition and the position of the B1, B2 and B3 domains, because these are known as identification criteria for *AFL* genes (McCarty et al., 1991; Giraudat et al., 1992; Suzuki et al., 1997; Nag et al., 2005; Lu et al., 2010). The AA composition of B1, B2, and B3 domains was analyzed by the WebLogo online (**Figures 2D, 3D** ^9^). We performed the intron-exon and position analyses of the *NF-YB* family genes by using their full-length DNA sequences (**Figure 4** and Supplementary Table S3).

To characterize *cis*-elements in the 5′ flanking region of *LAFL* genes, the 1.5 kb fragment containing promoter and 5′ UTR of six *AFL* genes and 40 *LEC1*-type genes were analyzed by PLACE (^10^this database is temporarily terminated now) and Matlnspector (Genomatix Software Suite ^11^) online (**Figures 3, 4** and Supplementary Table S5). Promoter sequences of *A. capillus-veneris LEC1* were cloned through genome walking (primers in Supplementary Table S4).

### Expression Analysis by qRT-PCR

To investigate the expression of *LAFL* genes in different vascular plants, the publicly available expression data as well as the expression data of *LEC1* homologs in *S. moellendorffii* and *A. capillus-veneris* (*SmoLEC1* expression data in Kirkbride et al., 2013, *AcaLEC1* unpublished expression data) were used to construct an expression heat map, where analyzed species include one monocot (rice), two eudicots (Arabidopsis and soybean), and one gymnosperm (*Picea abies*; Supplementary Figure S3). In addition, we chose *S. moellendorffii* and *A. capillus-veneris* as non-seed plant representatives using qRT-PCR to characterize *AFL* genes expression patterns at different developmental stages. With respect to sampling for these two species, *S. moellendorffii* roots, shoots, microphylls, strobili, and bulbils were collected in the field (Sichuan Province, voucher specimen was deposited in Peking University Herbarium, PEY). For *A. capillus-veneris*, samples were collected from plants cultivated in the greenhouse of Peking University (Voucher specimen was deposited in PEY). We chose roots, shoots, curled leaves, young leaves, fully developed leaves, immature sporangia, mature sporangia, immature gametophytes, reproductive gametophytes, and embryos as materials (Li et al., 2013). Total RNA of plant materials was isolated with Plant RNA Extraction Reagent (Invitrogen, USA) and purified with an RNeasy Mini kit according to the manufacturer’s instructions (Qiagen, Germany). The RNA was then converted to cDNA by reverse transcription with FastQuant RT Kit (Tiangen, China). The qRT-PCR was performed on an Applied Biosystems 7500 Real-Time PCR System (ABI) using cDNA templates mixed with primers (Supplementary Table S4) and SYBR^®^ Premix Ex Tax Mix (Takara, Japan). *SmoACTIN* and *AcaACTIN* were selected as the internal standard gene (primer sequences in Supplementary Table S4). Relative expression was calculated via delta-delta threshold method (2^-ΔCT^; Livak and Schmittgen, 2001). Results were summarized as means ± SE of three biological repeats.

## Results

### Phylogenetic Analysis of *LAFL* Genes

The sequence retrieval and phylogenetic analysis of the B3 gene superfamily showed that no *AFL* sequences were found in Chlorophytes and Charophytes (Supplementary Figure S1 and Table S1). The B3 domain of *AFL* genes is highly conserved in seed plants (Supplementary Table S2). According to phylogenetic analysis of *AFL* genes in land plants, the cluster of bryophytes and vascular plants can be recognized although they were not strongly supported in the tree (**Figures 1A, 2A**).

Phylogenetic analysis of bryophyte *AFL* genes showed them to form two clades, clade I and clade II (**Figure 1B**). Clade I is composed of sequences from liverworts and mosses, while clade II has sequences from liverworts, mosses and hornworts. Mosses have many more *AFL* gene homologs than do liverworts or hornworts (**Figure 1B** and **Table 1**).

In the phylogenetic tree of vascular plant *AFL* genes, three major clades, clade I (lycophyte *AFL* clade), clade II, and clade III (*ABI3* clade) can be recognized (**Table 1** and **Figure 2B**). Clade I is composed of all lycophyte *AFL* genes including those of *LEC2* type and *ABI3* type (**Figure 2B**). All monilophyte *ABI3* genes cluster with seed-plant *ABI3* genes as a clade with strong support (clade III) (**Figure 2B**), while monilophyte *LEC2* type genes group with the remaining seed plant *LEC2* and *FUS3* genes in clade II (**Figure 2B**). These results indicated that lycophyte *AFL* clade represents an ancient lineage of *AFL* gene family, and monilophyte *AFL* genes are more closely with those of seed plants. In the clade II, we can find five gene types with strong support, i.e., *LEC2* type, *FUS3* type, *LFL1* type, *PabFL* type, and *IDEF* type, respectively (**Figures 2B,C**). In the genome of the gymnosperm *P. abies*, there were five *AFL* sequences, and three of them (*PabAFLA/B/C*) are associated with *FUS3* and *LFL1* types. In the “early diverging” angiosperm *Amborella trichopoda* genome (Albert et al., 2013), there were three *AFL* sequences, none of them of *FUS3* type. One *ABI3* gene was clustered in the seed plant *ABI3* type clade strong support, one was clustered with the *LEC2* type sequences and the other was clustered in the *IDEF* clade.

Phylogenetic analyses of the *NF-YB* family showed that *LEC1*-type genes formed a clade and were only present in vascular plants (**Figure 4** and Supplementary Figure S3, Table S3), in agreement with previous results (Xie et al., 2008). The number of *LEC1*-type genes in lycophytes, monilophytes, conifers, “early diverging” angiosperms, monocots, and eudicots averages 1.0, 1.0, 2.0, 1.0, 2.0, and 2.7 per species, respectively (**Table 1**). *AcaLEC1* of the fern *A. capillus-veneris* was cloned and identified by this study for first time.

### Gene Structure Analysis of *LAFL* Genes

Domain structure analysis of bryophyte *AFL* genes showed that clade I genes only have one B3 domain in the C-terminal. This structure is similar to B3 genes in green algae. In clade II, *ABI3* type genes were easily recognized by the A domain, B1 (about 30 AA), B2 (about 14 AA) and B3 (about 100 AA) domain, while *ABI3*-like genes lack of the A domain. *LEC2* type genes had one unstable B2 domain in middle position and B3 domain in C-terminal. Notably, liverworts only had the clade I and *ABI3*-like *AFL* genes. We did not find *FUS3* type genes in bryophytes (**Figures 1C,D**), suggesting that *FUS3* may not arise. Domain structure analysis of vascular plant *AFL* genes showed that there were nine structure types in three clades with specific B2 domain AA characteristics (**Figure 2D**).

Our analyses of *NF-YB* gene sequences revealed that: (1) the *NF-YB* sequences restricted to algae are of the intron-rich type; (2) the liverwort *Marchantia polymorpha* contains both an intron-rich and an intron-less *NF-YB* gene; (3) six sequences occur in moss *Physcomitrella patens* including both intron-rich and intron-less ones; (4) *LEC1*-type genes, belonging to intron-less *NF-YB* genes, were only found in vascular plants. All those findings suggest that the intron-less type of *NF-YB* genes was derived from the intron-rich ones through gene duplication and intron loss in early land plants (**Figure 4C, Table 1**, and Supplementary Figure S3).

### *Cis-*Element Prediction of *LAFL* Genes

According to comparative analysis of *cis*-elements in regulatory region of *AFL* gene pairs between the seed plant Arabidopsis and the non-seed plant *S. moellendorffii*, we found that there was CCAAT-box in *AthABI3, AthFUS3, AthLEC2, SmoAFLB*, and *SmoAFLC* (**Figure 4** and Supplementary Table S5). By *cis*-elements prediction in the promoter region of 40 *LEC1*-type genes, we found that (1) *LEC1* had more *cis*-elements than do *L1L* genes in seed plants; (2) there is not a significant difference in *cis*-element components in *LEC1* genes from seed and non-seed plants: almost all the *cis*-elements identified in Arabidopsis can be found in the *LEC1* promoter of *S. moellendorffii* and *A. capillus-veneris* (**Figure 4B** and Supplementary Table S5).

### Expression Pattern Analyses of *LAFL* Genes

*LAFL* gene expression was restricted to seed development in Arabidopsis but occurs both in maturing seeds and inflorescences in other species, e.g., soybean, rice, and maize (**Figure 4D** and Supplementary Figure S4). For *LAFL* genes of *P. abies* (a gymnosperm), they were mainly expressed in leaves and cones. *SmoLEC1* and *AcaLEC1* were each only expressed in strobili and mature sporangia, and were not detected in tissues undergoing embryogenesis (**Figure 4D**).

The qRT-PCR results showed that the mRNA levels of *SmoAFLA* (lycophyte *ABI3* type) were nearly identical across organs (roots, shoots, microphylls, strobili, and bulbils) of *S. moellendorffii*. The levels of mRNA of *SmoAFLB* and *SmoAFLC* (lycophyte *LEC2* type) were higher in strobili than other organs. In *A. capillus-veneris* (a fern) the levels of mRNA of *AcaAFLA* (one monilophyte *ABI3* type) were higher in shoots and mature sporangia than other organs. The mRNA of *AcaAFLB* (another monilophyte *ABI3* type) and *AcaAFLC* (monilophyte *LEC2* type) were only detected in mature sporangia (**Figure 3** and Supplementary Figures S2, S4).

## Discussion

### LAFL Network and Seed Maturation

Previous studies showed that many genes are involved in seed maturation (Goldberg et al., 1994; Harada, 1997; Radoeva and Weijers, 2014). Among them, the *AFL* family of B3 transcription factors (TFs) and the *LEC1*-type of *NF-YB* TFs, which together form LAFL regulatory network, are considered to play key roles in seed maturation. Although there were studies on the evolution of *LEC1*-type genes (Xie et al., 2008), *AFL* genes (Li et al., 2010; Carbonero et al., 2016), this study presents a comprehensive analysis of *LAFL* genes by integrating their phylogeny, gene structure, *cis*-elements and expression patterns together for a better understanding of the evolution of seed maturation programs during plant evolution.

### Evolution and Function Differentiation of *AFL* Genes

According to our extensive phylogenic and gene structure analyses, *LEC2* type and *ABI3* type genes evolved in a common ancestor of bryophytes and vascular plants, and their gene structure is very conservative. However, *FUS3* type genes were only found in seed plants (**Figures 1, 2**), suggesting that *FUS3* genes originate relatively late in the *AFL* family.

In embryophytes, *LEC2* type genes had one B2 domain in a middle position and a B3 domain in the C-terminal. In the seedless species *S. moellendorffii* (lycophyte) and *A. capillus-veneris* (fern), the expression pattern of *LEC2* type genes (*SmoAFLB, SmoAFLC*, and *AcaAFLC*) was restricted to shoots (*S. moellendorffii*) and maturing spores (both *S. moellendorffii* and *A. capillus-veneris*; **Figure 3**). In the “early diverging” angiosperm *Amborella trichopoda*, there were three *AFL* genes. One of them is of *ABI3* type, and the other two are *LEC2* type and *IDEF* type, respectively. Interestingly, *IDEF* type genes were identified only from monocots, and have only B3 domain in C-terminal (Kobayashi et al., 2007), which is different from LEC2 gene structure (**Figure 2C**). In rice, *OsaIDEF* transcripts are constitutively present in roots, leaves, inflorescences, and seeds. In eudicots, *LEC2* plays central roles in seed embryogenesis and morphogenesis (**Figure 2** and Supplementary Figure S4). All these data suggest that *LEC2* and *IDEF* type genes diverged very early, and *LEC2* type genes may be lost in monocots.

During the review of this manuscript, Carbonero et al. (2016) published their work on the AFL family. In agreement with our results, they suggest that the origin of the AFL family traces back to a common ancestor of bryophytes and vascular plants, and that this family has expanded in the angiosperms. However, due to different sampling regimes and sequence coverage, there are some different results between these two studies, especially relating to the evolution of *LEC2* genes. According to Carbonero et al. (2016), seven *LEC2* genes were described from three monocots, *Oryza sativa, Brachypodium distachyon* and *Hordeum vulgare* (all grasses), but the relationship of those seven genes with other *AFL* homologs needs to be verified; differences in gene structure, phylogenetic position, and expression pattern suggests that these may not be *LEC2* genes.

Considering *ABI3* genes of land plants, there is a clear evolutionary trajectory according to our study. Phylogenetically, monilophyte *ABI3* genes are more closely related to those of seed plants, rather than to lycophyte *ABI3* types. In *P. abies* (gymnosperm) and *Amborella trichopoda* (“early diverging” angiosperm), there was only one *PabABI3* and *AtrABI3* sequence, respectively. This may be due to the lack of a lineage-specific whole genome duplication (WGD) in these species (Albert et al., 2013; Nystedt et al., 2013). Expression patterns of *SmoAFLA* (*S. moellendorffii*, one lycophyte *ABI3* type) are more similar to those of bryophyte *ABI3* type genes, which are only expressed in vegetative tissues (**Figure 3**; Khandelwal et al., 2010). The expression of *AcaAFLA* and *AcaAFLB* (*A. capillus-veneris*, two monilophyte *ABI3* type genes) are found in shoots and spore maturation, which are consistent with that of *PabABI3* (*P. abies*) (**Figures 2B,C,E, 3**). This suggest the expression pattern of *ABI3* genes has slightly differentiated across major land plant lineages.

*FUS3* type genes appear to have originated relatively late because they are restricted to the seed plant clade. Three *PabAFL* sequences (*PabAFLA, B, and C*) from the gymnosperm *P. abies* belong to *Pab-FL* (*FUS3* and *LFL*) type clade, which is associated with *FUS3* type and *LFL* type. These finding, coupled with expression patterns of *PabAFLA/B/C* genes suggest that the *Pab-FL* type may represent ancestral *FUS3/LFL* gene function. There is no *FUS3* type member in *Amborella trichopoda*, which suggests that *FUS3* type genes likely originated in a common ancestor of seed plants and were subsequently lost in *Amborella*. In eudicots and monocots, *FL* genes are divided to *FUS3* type and *LFL* type, respectively. *OsLFL1*, involved in the photoperiodic flowering of rice and expressed exclusively in spikes and young embryos, is functionally similar to *AthFUS3* in Arabidopsis (Peng et al., 2008; Tiedemann et al., 2008). The *FUS3* type (found only in eudicots) and the *LFL* type (restricted to monocots) are clustered together with strong bootstrap support, and they have similar domain structure and functions (**Figures 2B,C,E, 3**).

### Evolution of the *LEC1-Type* Genes

As members of the LAFL network, *LEC1*-type genes are CCAAT-binding factors (CBFs), which are present in all eukaryotes (Forsburg and Guarente, 1989; Mantovani, 1999; Matuoka and Chen, 2002; Siefers et al., 2009; Dolfini et al., 2012). There is no clear correlation between expression patterns and the classification of *NF-YB* family genes with an exception of the *LEC1*-type genes, which are considered seed-specific (Stephenson et al., 2007; Salvini et al., 2012). Arabidopsis *LEC1*-type genes (*AthLEC1* and *AthL1L*) have significant functions at late stages of embryogenesis (Lotan et al., 1998; Kwong et al., 2003). Our phylogenetic analyses of the *NF-YB* gene family support some findings of previous studies, e.g., only one intron-rich type of *NF-YB* genes occurs in chlorophytes, the intron-less genes are derived from the intron-rich ones, and *LEC1*-type genes are restricted to vascular plants (Xie et al., 2008; Cagliari et al., 2014; **Table 1** and **Figures 4A,C**).

In addition, there are some new findings, e.g., only one copy of the intron-rich type of *NF-YB* genes is found in the alga *Klebsormidium flaccidum*, which is considered to be one of the closest relatives of land plants (Hori et al., 2014). The liverwort *Marchantia polymorpha*, one of the earliest diverged land plants (Rövekamp et al., 2016), has two copies of *NF-YB* genes in its genome, one of which is intron-rich and the other intron-less. The six copies found in the moss *Physcomitrella patens*, have been proven to originate from duplication events (Yang et al., 2005; Rensing et al., 2008; Xie et al., 2008). In addition, our analyses demonstrate that there is only one copy of *LEC1*-type genes in the genome of *S. moellendorffii* (lycophyte), *A. capillus-veneris* (fern), *P. abies* (gymnosperm), and *Amborella trichopoda* (“early diverging” angiosperm). These data support that *LEC1* and *L1L* genes result from the duplication of *LEC1*-type genes likely occurring after the origin of extant angiosperms (**Table 1** and **Figures 4A,C**).

### The *Cis-*Element Prediction and Co-expression of *LAFL* Genes

The LAFL network has been considered to play central roles in seed maturation, and *LAFL* genes regulate different facets of this developmental process by their interactions with up- and down-stream genes (Harada, 1997; Santos-Mendoza et al., 2008; Fatihi et al., 2016; González-Morales et al., 2016). The *cis*-element prediction shows that *LEC1* genes of seed plants and non-seed plants have similar *cis*-elements, suggesting the *LEC1-*type genes could be regulated by similar regulators (**Figure 4**). Among the *cis*-elements of *LEC1*, RYREPART and ABRE are thought to be very important for *LEC1* activity. The RYREPEAT is considered to be a RY-like element, and the binding site of the B3 domain (Braybrook et al., 2006; Mönke et al., 2012; Wang and Perry, 2013; Tang et al., 2015). The ABRE is functionally important in many ABA-regulated genes (Fan et al., 2015). Additionally, *LEC1*, as a subunit of the CCAAT-box binding factor (CBF), activates its downstream genes by the CCAAT-box element (Junker et al., 2012). According to the CCAAT-box element prediction of *AFL* genes in *S. moellendorffii*, there is a CCAAT-box element in the regulatory region of its *AFL* genes, e.g., *SmoAFLB* and *SmoAFLC* (**Figure 3**).

The findings presented in this study suggest that a partial LAFL network, consisting of *ABI3* and *LEC2* genes, arose in a common ancestor of land plants, and then became more complex with the occurrence of *FUS3* and *LEC1* genes. With evolution of vascular plants, *LAFL* network genes likely specify their co-expression in two different developmental processes, spore and seed maturation, respectively. The co-expression of *LAFL* genes in these two processes alone or simultaneously, which correspond to two reproductive structures, suggest that the biological process involved in spore maturation is similar to those of seed maturation.

## Author Contributions

J-DH analyzed data and drafted the manuscript. XL and C-KJ carried out the experiments. GW and CR provided some samples and analyzed sequences. G-YR designed the research.

## Conflict of Interest Statement

The authors declare that the research was conducted in the absence of any commercial or financial relationships that could be construed as a potential conflict of interest.

## Footnotes

1 https://pfam.xfam.org/family/PF02362#tabview=tab0

2 https://phytozome.jgi.doe.gov/pz/portal.html

3 congenie.org

4 www.plantmorphogenesis.bio.titech.ac.jp/~algae_genome_project/klebsormidium/index.html

5 https://db.cngb.org/blast4onekp/

6 www.ncbi.nlm.nih.gov

7 http://mafft.cbrc.jp/alignment/server/

8 http://tree.bio.ed.ac.uk/software/figtree/

9 weblogo.berkeley.edu

10 sogo.dna.affrc.go.jp/

11 https://www.genomatix.de/solutions/genomatix-software-suite.html
