# Supplementary material for: Evolutionary Analysis of the LAFL Genes Involved in the Land Plant Seed Maturation Program
Source: Front Plant Sci. 2017 Apr 4;8:439. doi: 10.3389/fpls.2017.00439 (PMC5379062; doi:10.3389/fpls.2017.00439)
Supplement: Supplementary file 1 [file Data_Sheet_1.DOCX]

Supplementary materials

Figure S1. ML tree showing the phylogenetic relationships of *B3* superfamily by phylogenetic analyses of 730 B3 domain sequences from 11 taxa (species name list see Table S1). *AFL* family is highlighted with blue block and star.

Figure S2. Comparison of amino acid sequences of *AthABI3*, *AthLEC2*, *AthFUS3*, *AcaAFLA*, *AcaAFLB*, *AcaAFLC*, *SmoAFLA*, *SmoAFLB* and *SmoAFLC*. The alignment is made by the MAFFT program (See materials and methods). The A, B1, B2 and B3 domains are indicated by the black, blue, green and red box, respectively.

Figure S3. ML tree showing the phylogenetic relationships of *NF-YB* family by phylogenetic analyses of 263 sequences from 29 taxa (species name list see Table S3). *LEC1*-type clade is highlighted with purple block and star.

Figure S4. Expression profile of (A) *LEC1-type* genes and (B) *AFL* genes in *O. sativa*, *G. max*, *A. thaliana* and *P. abies*. The first three expression analyses are run out using Genevestigator software (https://www.genevestigator.com). For *P. abies*, the relative values of expression in various organs were acquired from ConGenIE (<http://congenie.org)>. (C) *OsaIDEF1* expression pattern and (D) *OsaIDEFH* expression pattern (data from rice eFP Browser, http://bar.utoronto.ca/efprice/cgi-bin/efpWeb.cgi).
